# Stillbirth differences according to regions of origin: an analysis of the German perinatal database, 2004-2007

**DOI:** 10.1186/1471-2393-11-63

**Published:** 2011-09-21

**Authors:** Anna Reeske, Marcus Kutschmann, Oliver Razum, Jacob Spallek

**Affiliations:** 1 Department of Epidemiology & International Public Health, School of Public Health, Bielefeld University, P.O. Box 10 01 31, D-33501 Bielefeld, Germany; 2 Bremen Institute for Prevention Research and Social Medicine (BIPS), Department of Prevention and Evaluation, University of Bremen, Achterstraße 30, D-28359 Bremen, Germany; 3 BQS Institute for Quality and Patient Safety, Department of Medical Biometry, Kanzlerstraße 4, D-40472 Düsseldorf, Germany

**Keywords:** stillbirth, region of origin, relative differences, risk factors

## Abstract

**Background:**

Stillbirth is a sensitive indicator for access to, and quality of health care and social services in a society. If a particular population group e.g. migrants experiences higher rates of stillbirth, this might be an indication of social deprivation or barriers to health care. This study examines differences in risk of stillbirth for women of different regions of origin compared to women from Germany in order to identify high risk groups/target groups for prevention strategies.

**Methods:**

We used the BQS dataset routinely compiled to examine perinatal outcomes in Germany nationwide. Participation of hospitals and completeness of data has been about 98% in recent years. Data on all live births and stillbirths were obtained for the period 2004 to 2007 (N = 2,670,048). We calculated crude and stratified mortality rates as well as corresponding relative mortality risks.

**Results:**

A significantly elevated stillbirth rate was found for women from the Middle East and North Africa (incl. Turkey) (RR 1.34, CI 1.22-1.55). The risk was slightly attenuated for low SES. An elevated risk was also found for women from Asia (RR 1.18, CI 1.02-1.65) and from Mediterranean countries (RR 1.14, CI 0.93-1.28). No considerable differences either in use and timing of antenatal care or preterm birth and low birthweight were observed between migrant and non-migrant women. After stratification for light for gestational age, the relative risk of stillbirth for women from the Middle East/North Africa increased to 1.63 (95% CI 1.25-2.13). When adjusted for preterm births with low birthweight, women from Eastern Europe and the Middle East/North Africa experienced a 26% (43%) higher risk compared with women from Germany.

**Conclusions:**

We found differences in risk of stillbirth among women from Middle East/North Africa, especially in association with low SES and low birthweight for gestational age. Our findings suggest a need for developing and evaluating socially and culturally sensitive health promotion and prevention programmes for this group. The findings should also stimulate discussion about the quality and appropriateness of antenatal and perinatal care of pregnant women and newborns with migrant backgrounds.

## Background

Although stillbirth occurs rather rarely it is one of the more devastating events in obstetrics and a sensitive indicator for the quality of health care, living conditions and inequity in a society. If a particular population group, such as migrants experiences higher rates of stillbirth than the non-migrant population, this might be an indication of social deprivation or access to health care barriers.

Migrants tend to be more vulnerable in terms of morbidity and mortality patterns compared to non-migrant populations, influenced e.g. by socioeconomic factors, barriers to prevention and health care offers, as well as migration and cultural related factors [1,2].

Recent published worldwide estimates of stillbirth rates showed a decline from 22.1 stillbirths per 1000 births in 1995 to 18.9 in 2009, with the highest rates being observed in south Asia and sub-Saharan Africa [3]. It is, however, also a topic of high public health relevance in high-income countries because of the disparities in stillbirth rates among different ethnic groups in these societies [4]. In Europe and the USA, several studies have shown disparities in stillbirth, perinatal and infant mortality among ethnic groups compared with the host population of each country [5-11]. In European countries, risks of stillbirth are elevated especially for women from non-western countries, e.g. from Turkey, Morocco, Pakistan, Somalia and Ghana [5-7]. A recent systematic literature review on differences in stillbirths and infant deaths between migrant and non-migrant populations in industrialized countries however shows inconsistent results. Half of the studies reported worse mortality outcomes for migrants, one third did not find considerable differences and a few found better mortality outcomes for migrants compared with the non-migrant population [12]. Furthermore, the distribution of risk factors for stillbirth, such as preterm birth [13-15], low birthweight [16-18], congenital anomalies [19,20], and inappropriate use of antenatal care [21-23], varies between ethnic groups in European countries.

In Germany, nationwide analyses on differences in stillbirth among migrants and non-migrants are lacking. Despite a positive trend in recent decades, there are indications that infant mortality among non-Germans is still higher than among Germans, [1]. Regional studies confirm this finding [24,25]. Women with Turkish origin and immigrant women with a short duration of stay seem to be mainly affected [1,25,26]. Some studies also indicate differences between women with migrant backgrounds compared with women without migrant backgrounds in terms of certain risk factors for stillbirth [27-30]. Nevertheless, nationwide analyses on risk differences of stillbirth for different ethnic groups in Germany are still missing.

We compared the risk of stillbirth among women of different regions of origin with that of women without migrant backgrounds. Our aim was to identify high risk groups/target groups for prevention strategies in order to reduce inequalities in stillbirth and associated factors. The present study is the first in Germany to use nationwide perinatal data for the investigation of variations in the risk of stillbirth in relation to maternal migrant background.

## Methods

### Database

We used anonymous data from the nationwide perinatal database maintained by the German National Agency for Performance Measurement in Health Care [Bundesgeschäftsstelle Qualitätssicherung gGmbH (BQS), since 2010: BQS Institute for Quality and Patient Safety]. From 2001-2009, the BQS was responsible for the development and implementation of comparative quality assurance in German hospitals. For this purpose, the BQS collected basic data and quality indicators from all hospitals in Germany. Participation in the quality assurance program is compulsory for all hospitals by law. Hence, the BQS database contains complete data concerning hospital-related processes of medical and nursing care. Participation of hospitals and completeness of data was about 98% in recent years [31]. All phases of the study were subject to the strict data protection regulations of the BQS and the German law. The study was performed in accordance with the guidelines for Good Scientific and Good Epidemiological Praxis of the German Society for Epidemiology (DGEPI 2008).

Ethical approval was not needed for our study as we fulfilled the criteria of the "Good Practice in Secondary Data Analysis" (GPS) of the German epidemiological societies. According to this guideline we observed all data protection provisions for secondary data analysis. Only anonymised data were used. Hence a re-identification of persons was not possible and no informed consent of participants was necessary.

Quality assessment in the field of obstetrics is based on a nationally standardized electronic data entry form which contains items concerning socio-demographic data of the mother, course of current pregnancy, delivery and birth outcomes, and health of the newborn. The perinatal database includes only live births and stillbirths born in a hospital, but no spontaneous abortions and home deliveries.

The perinatal data entry form is filled in by the staff of the labour room. Pregnancy-related information is obtained from the maternity care records each mother is supposed to carry during pregnancy, and birth-related information is obtained from the hospital records. Afterwards, data is submitted electronically to the Agency for Performance Measurement in Health Care (Landesgeschäftsstelle für Qualitätssicherung [LQS]) in the federal state in which the hospital is located. The LQSs ensure anonymization and internal validation of the data and submits them to the BQS.

We obtained pregnancy and birth-related data for all live- and stillbirths during the time period 2004 to 2007 (N = 2,670,048).

### Outcome

The main outcome measure was the relative risk of stillbirth for women from different regions of origin compared with women without migrant backgrounds. In Germany, the definition of stillbirth is a birth without vital signs after delivery and with a birthweight of at least 500 g. Births with a birthweight of less than 500 g were excluded from the analyses. Stillbirth rate was defined as the number of stillbirths during the period from 2004 to 2007 per 1,000 live- and stillbirths during the same period. An external validation procedure with population data of the Federal Statistical Office revealed that completeness of reporting of stillbirths was about 90%. Differential recording of stillbirths is improbable because stillbirths are recorded directly after delivery.

### Region of origin

In the German perinatal database, migrant background is assessed by the mother's region of origin, grouped in six bands: (1) Germany, (2) Middle and Northern Europe, North America, (3) Mediterranean countries, (4) Eastern Europe, (5) the Middle East and (6) Asia. An overview of countries in the different bands can be found in the Additional file 1 Table S1. Germany's largest migrant group by nationality, the Turkish migrants, is assigned to the "Middle East", together with Afghanistan, Pakistan, and the Arab countries of North Africa. A separation of countries within a band is not possible. The group "other countries" was excluded from the analyses as there is no information on which countries are included in this band. We did not obtain information on the manner and consistency concerning the assessment of "region of origin" in German hospitals. Thus we cannot exclude the possibility that hospital staff completing the perinatal sheet used concepts and definitions on migrant background other than region of origin (e.g. nationality). In the present paper, we referred to women originating from Germany as "women without migrant backgrounds" or "women from Germany", although this might be imprecise.

### Stratification factors

#### Socio-demographic and lifestyle-related factors

The socioeconomic status of mothers is insufficiently measured in the database. The variable "mother's occupation" was the only one available as proxy for her socioeconomic status. We classified "housewife" and "un-/skilled worker" into low SES, "low-level office worker" and "currently being a trainee/student" into middle SES and "middle/high-level office worker" into high level SES. Nevertheless, it is a rather insufficient indicator for the SES, because subsidiary details concerning the occupation and income of the newborn's father are not included in the database. We assessed "being single (yes/no)" as a further available proxy for an unfavourable socioeconomic situation. Maternal age was classified in five groups (< 18, 18-29, 30-34, 35-39, > 39) and parity in three groups (0, 1, ≥ 2). The variable "smoking after awareness of pregnancy" was grouped in three categories (yes/no/missing information).

#### Obstetric factors

Concerning obstetric determinants, the following information was included: low birthweight (< 2500 g, ≥ 2500 g), gestational age in weeks (< 28, 28-31, 32-36, ≥ 37) and congenital anomalies (yes/no). Preterm delivery is often associated with low birthweight. In order to explore interaction effects of gestational age & birthweight on the stillbirth risk among women with and without migrant background, we defined an interaction variable grouped in four categories:

(1) preterm delivery (< 37 gestational weeks) combined with low birthweight (< 2500 g),

(2) preterm delivery (< 37 gestational weeks) combined with normal birthweight (≥ 2500 g), (3) term delivery (≥ 37 gestational weeks) combined with low birthweight (< 2500 g),

(4) term delivery (≥ 37 gestational weeks) combined with normal birthweight (≥ 2500 g).

#### Antenatal care

Use of antenatal care was assessed on the basis of number of antenatal care visits and the timing of the first visit during pregnancy. According to the BQS definition, number of antenatal care visits was classified in (1) 0-4 visits (considered "insufficient care"), (2) 5-7 visits, (3) 8-11 visits, (4) > 11 visits (considered "above-average care"). The timing of first antenatal visit in gestational weeks was grouped in four categories: (1) < 9 gestational weeks, (2) 9-12 weeks, (3) 13-16 weeks, (4) > 16 weeks. Furthermore, the number of ultrasounds and the timing of first ultrasound during pregnancy was also assessed according to the BQS definition. The number of ultrasounds was classified into (1) 0-2, (2) 3-4 (according to the German maternity guideline considered as standard for low risk pregnancies), (3) > 4. The timing of first ultrasound in gestational weeks was grouped into (1) 0-8, (2) 9-12, (3) 13-16 and (4) > 16 weeks.

#### Maternal co-morbidities

Maternal co-morbidities during pregnancy are assessed as pregnancy risk factors. We included pre-eclampsia, eclampsia, placenta praevia (antenatal) and placenta praevia (during birth) as important risk factors for stillbirth, classified into binary variables (yes/no).

### Statistical analysis

Firstly, we tested differences in socioeconomic, pregnancy and delivery related characteristics between the migrant groups and the German reference group using chi-square tests.

Secondly, we calculated crude stillbirth rates for each group by region of origin. Stillbirth rates were calculated as all stillbirths in a group divided by all live- and stillbirths in the same group. We also performed a stratified analysis by each afore mentioned factor to assess its influence on differences in stillbirth rates between women of different region of origin and women without migrant backgrounds.

Thirdly, we calculated crude and stratified relative mortality risks and 95% confidence intervals. These expressed the relative risk of stillbirth for women from each foreign region of origin compared with women without migrant backgrounds (reference group).

All statistical analyses were performed using the Statistical Package of Social Science version 16.0 (SPSS Inc., Chicago, IL, USA).

## Results

During the period 2004-2007 there were 2,623,064 livebirths and stillbirths in Germany of which 504.043 (18.9%) were to women from foreign regions of origin. Of the mothers, 37,854 originated from Middle and Northern Europe or North America, 85,874 from Mediterranean countries, 138,217 from Eastern Europe, 164,341 from the Middle East or North Africa (incl. Turkey) and 30,773 from Asia. Migrants grouped in the category "other countries" were excluded from the analysis (n = 46,984). Table 1 details the numbers of births and selected characteristics of mothers and their newborns by region of origin. Further characteristics can be found in Additional file 2, Table S2.

**Table 1:** Characteristics of study population by mother's region of origin (in %), 2004-2007, (N = 2,623,064)

	Germany	Middle and Northern Europe,	Mediterranean	Eastern Europe	Middle East,	Asia	p value*
		North America			North Africa		
**Number of births**	2,166,005	37,854	85,874	138,217	164,341	30,773	
**Single status**							
No	76.7	83.6	86.1	85.4	90.1	83.9	< 0.0001
Yes	14.9	9.0	6.8	8.4	4.0	8.5	
Missing	8.4	7.4	7.0	6.1	5.9	7.5	
**Social status**							
Low	31.8	45.4	59.8	56.1	71.4	62.0	< 0.0001
Middle	37.8	24.1	16.3	21.6	9.6	13.4	
High	13.5	12.4	4.4	5.2	2.2	4.4	
Missing	16.9	18.1	19.6	17.1	16.8	20.3	
**Maternal age (years)**							
< 18	0.7	0.3	0.6	0.5	0.3	0.2	< 0.0001
18-29	43.4	42.3	55.8	60.6	59.2	45.0	
30-34	31.2	32.3	28.2	26.2	25.8	30.8	
35-39	20.3	20.3	12.7	10.4	11.8	19.4	
≥ 40	4.4	4.8	2.6	2.4	2.8	4.7	
**Maternal smoking (during pregnancy)**							
No	72.9	72.7	67.7	71.0	73.3	79.3	< 0.0001
Yes	10.9	6.2	11.2	7.3	9.2	1.8	
Missing	16.2	21.1	21.2	21.7	17.4	18.9	
**Parity**							
0	42.5	35.9	32.9	35.7	29.3	36.0	< 0.0001
1	32.1	33.4	32.0	32.1	29.4	33.7	
≥ 2	25.4	30.7	35.0	32.2	41.3	30.3	
**Multiple births**							
Singletons	96.6	96.7	96.9	97.3	97.0	97.8	< 0.0001
Twins or more	3.4	3.3	3.1	2.7	3.0	2.2	
**Gestational age (weeks) & birth weight (g)**							
< 37 & < 2500	4.5	4.1	4.1	3.8	4.3	4.2	< 0.0001
≥ 37 & < 2500	2.7	2.3	2.6	2.0	2.6	2.9	
< 37 & ≥ 2500	1.7	1.6	1.5	1.6	1.6	1.5	
≥ 37 & ≥ 2500	91.1	92.0	91.7	92.6	91.5	91.4	
**Number of antenatal visits**							
0-04	1.4	2.6	2.8	3.1	2.2	3.3	< 0.0001
5-07	4.3	6.9	7.9	7.8	7.8	9.0	
8-11	43.1	42.3	47.2	47.0	50.0	49.4	
≥ 12	43.1	37.9	33.0	34.7	32.9	29.4	
Missing	8.1	10.3	9.2	7.4	7.2	8.9	
**First antenatal visit (gestational week)**							
< 9.	46.5	40.3	42.2	39.7	44.9	35.8	< 0.0001
9.-12.	37.5	34.7	35.6	36.9	36.0	38.0	
13.-16.	5.7	7.7	7.7	8.8	7.6	9.5	
≥ 17.	3.1	8.0	5.9	8.2	5.0	9.3	
Missing	7.2	9.3	8.6	6.4	6.5	7.4	
**Congenital anomalies**							
No	99.0	98.9	98.8	98.9	99.0	99.0	< 0.001
Yes	1.0	1.1	1.2	1.1	1.0	1.0	

* p value for a chi-square test

Compared with women without migrant backgrounds, women with migrant backgrounds were of younger age at delivery, more often multiparous, and less often single. The proportion of single women was lowest among women from the Middle East and North Africa. They also showed the highest proportion of mothers with a low social status (71.4%).

In our sample, women with and without migrant backgrounds showed no substantial differences in the number and timing of antenatal care visits. In all groups about 40% of pregnant women made sufficient use of antenatal care (8-11 visits). The proportion of women with insufficient care (0-7 visits) was low (under 3%). About 80% of pregnant women attended their first visit prior to gestational week 13, and nearly half of them attended before gestational week 9. Only women from Eastern Europe and Asia showed a higher prevalence of having their first visit rather late in pregnancy (after gestational week 12) and receiving only 0-4 visits (3.1 and 3.3 respectively, p < 0.0001).

The same applied to the number and timing of ultrasound examinations. About 50% of migrant mothers had an appropriate number of ultrasound scans (3-4 scans) and about 70% had their first ultrasound within the first trimester, compared to 49.9% and 84%, respectively, among German mothers.

We could not find relevant differences between women with and without migrant backgrounds for preterm delivery, low birthweight, smallness for gestational age (SGA), congenital anomalies and eclampsia during delivery. Pre-eclampsia was more prevalent among mothers from Germany (2.5%, p < 0.0001) compared to 1.3% among mothers from the Middle East and North Africa and 1.2% among Asian mothers. Although the proportion was rather low, compared to the other groups Asian women experienced a higher risk of placenta praevia during pregnancy or birth (Additional file 2 Table S2).

Additional file 2 Table S3 shows the number of stillbirths and crude stillbirth rates by mother's region of origin as well as relative mortality risks for migrant women compared with women from Germany. During the time period 2004 to 2007, a significantly elevated stillbirth rate was found for women originating from the Middle East and North Africa (incl. Turkey) in comparison to German women (RR 1.34, CI 1.22-1.55). A slightly higher risk was found for women from Asia (RR 1.18, CI 1.02-1.65). Women from Mediterranean countries had a slightly increased risk for stillbirth, but this increase was not significant (RR 1.14, CI 0.93-1.28). Relative risks of stillbirth for women from Middle and Northern Europe, North America and Eastern Europe were not elevated.

### Stratified analyses

In stratified analyses, we examined the effect of the previously described factors on the relative differences in the relative risk of stillbirth for women with migrant backgrounds compared with women without migrant backgrounds (Additional File 2 Table S3), in order to identify possible confounders or effect modifications.

The relative risk of stillbirth was modified after adjustment for social status in all migrant groups. A significantly increased relative risk was only obvious among women from Middle East and North Africa with a low social status (RR 1.29, 95% CI 1.17-1.42). Amongst those with high social status, the relative risk was also elevated, but did not reach statistical significance. The number of stillbirths in this group was small.

Prior to gestational week 28, the risk of stillbirth for women with migrant backgrounds was mostly lower compared to women from Germany. In the time period 28 to 31 gestational weeks however it was significantly increased for Middle East and North African women (RR 1.34, 95% CI 1.13-1.59) and between 32 and 36 gestational weeks it was significantly increased for all migrant women compared to the German population, except for women from Middle and Northern Europe and North America (statistically significant at 5% level). The risk for women from the Middle East and North Africa was 70% higher during this period.

Adjusting for birthweight increased the relative risk in the birthweight groups under 2,500 g (low birthweight) significantly among women from Eastern Europe and those from the Middle East and North Africa.

Concerning the interaction of preterm birth and low birthweight, the risk of stillbirth for women from the Middle East and North Africa compared with the majority population was highest after 37 gestational weeks combined with low birthweight (RR 1.63, 95% CI 1.25-2.13). Women from Eastern Europe and from the Middle East and North Africa respectively experienced a 26% and 43% higher risk of stillbirth before 37 gestational weeks combined with low birthweight (both statistical significant at a 5% level).

Among women who attended antenatal care before week 9 of their pregnancy, the relative risk of stillbirth was significantly higher for migrants from the Middle East and North Africa, for Mediterranean countries and for Asia compared to women from Germany (RR 1.40, 1.37 and 1.48 respectively), but it was lower among those who attended late. The trend was similar when the number of antenatal visits was analyzed: amongst women with a substandard number of visits, women with migrant backgrounds showed a lower relative risk of stillbirth compared with women without migrant backgrounds. However, amongst women attending more than 11 visits, women from the Middle East and North Africa showed a 41% higher risk compared with women without migrant backgrounds. The relative risks of stillbirth adjusted for congenital anomalies remained stable (Additional file 2 Table S3).

## Discussion

### Interpretation of differences in stillbirth risk

The present study is the first in Germany to use nationwide perinatal data for the investigation of variations in the risk of stillbirth in relation to maternal migrant background.

Using the German perinatal database, we found relative differences in the risk of stillbirth for women of different regions of origin compared with women from Germany. Women from the Middle East and North Africa (including immigrant women from Turkey) showed the highest risk. After adjustment for maternal characteristics and obstetric factors, the higher risk of stillbirth among women from the Middle East and North Africa was partly attenuated for example when stratified for mother's occupation. However the overall risk remained stable for other characteristics. In contrast to prior studies we did not find any considerable differences between women originating from foreign regions of origin compared to women from Germany in terms of obstetric factors that might explain the higher stillbirth rates in some migrants groups, e.g. in timing and number of antenatal care visits as well as preterm birth and low birthweight. As the factors analysed did not confound or modify the association between migrant background and increased risk of stillbirth in the majority of stratified analyses, migrant status may have an independent effect on the risk of stillbirth. As migrant background might be a proxy for other underlying factors such as cultural factors, social deprivation, access barriers etc., further studies should focus on these factors.

The increased relative risk of stillbirth among women with migrant background was slightly mitigated after stratification for socio-demographic factors, such as single status, low social status, young age of mother and high parity (Tables 2) but it was still elevated compared with women from Germany. These results are in line with a Danish study, where a higher risk of stillbirth for Turkish and Pakistani women was not associated with their higher parity, lower educational level and lower household income compared to the Danish women [6].

As the variables available for SES were insufficient in this study we have to interpret our results very carefully. In addition, the population groups in each band are very heterogeneous e.g. in terms of socioeconomic status, making it difficult to examine associations between migrant background, SES and risk of stillbirth. Compared to other regions the Middle East and North Africa had the highest proportion of women with low SES. These women also experienced a higher risk of stillbirth. This might point to the importance of socioeconomic factors e.g. education and income as well as socioeconomic-related factors e.g. integration level and working possibilities of migrants when looking at stillbirth risks. This is in line with a study from the Netherlands, where higher infant mortality rates among Turkish migrants compared to women from the Netherlands were partly explained by socioeconomic and demographic factors [32]. Consequently, women from the Middle East and North Africa in Germany comprise an important target group for future prevention strategies in the field of pregnancy care.

Several studies have reported an insufficient and late use of antenatal care among migrant women compared to non-migrant women [21-23,33]. Causal factors identified include language barriers, lack of knowledge concerning antenatal care, psychosocial factors and lower education. Our findings point in a different direction: we did not find considerable differences in the number and timing of antenatal care between women with and without migrant backgrounds, both with livebirths and stillbirths. The majority of women in all groups attended antenatal care before gestational week 9 and recorded eight to eleven visits. This indicates that *access *to health care services is not worse for the majority of migrant women in Germany, at least during pregnancy, and the majority seems to be informed about the appropriate timing and number of antenatal visits. What we found was an excess risk of stillbirth for women from the Mediterranean countries and Asia amongst those who attended antenatal care prior to gestational week 9 and for women from the Middle East and North Africa, who attended antenatal care very early and received a sufficient number of visits.

In contrast, previous studies based on regional data of the German perinatal database found a less frequent use of antenatal care, in particular for single women with migrant backgrounds [34], women from Eastern Europe and Mediterranean countries [27,30] and a later use of antenatal care among women from the Middle East [29], from Eastern Europe and from Mediterranean countries [30]. In addition, regional differences in the analysis of BQS data indicate that our findings may vary between federal states as well as urban and rural areas within single federal states in Germany. Comparisons of results between the previous German studies and our study are difficult because of the variations in defined number, timing of antenatal care and differences in the studied migrant groups. Nevertheless, we cannot rule out the possibility of variations in use and timing of antenatal care according to particular countries of origin and regional levels in Germany. Hence, further analyses of risk differences in stillbirth and its determinants on regional level and for precisely defined migrant groups are necessary.

In our study, women with migrant background with only a few or no antenatal visits, or who attended very late, showed a lower risk of stillbirth compared with women without migrant background with similar utilization patterns. Women with migrant background give birth in younger years and have a higher parity compared with women without migrant background (Table 1 and 2). It can be assumed that the women with migrant backgrounds are a selection of young women who already gave birth to several children without severe complications and altogether show a low risk profile.

Several studies from Europe and the USA showed a higher maternal risk of preterm birth and low birthweight for migrant women [13-18]. In contrast, there are several studies showing a lower risk for migrants despite risk factors such as higher parity and lower socioeconomic status [11,27,28]. In line with these findings we did not find differences in preterm birth and low birthweight prevalence between women of different regions of origin and women from Germany. When looking at the risk of stillbirth adjusted for birthweight, women from Eastern Europe and from the Middle East and North Africa experienced a significantly higher risk. Our findings also suggest that birthweight relative to the gestational age seems to have an influence on the differences in risk of stillbirth: a low birthweight combined with a regular gestational age ("small or light for dates") contributed to a higher risk of stillbirth, especially for women from the Middle East and North Africa compared to women from Germany. This might be an indication for a higher stillbirth risk among these women associated with intrauterine growth retardation (IUGR) or fetal malnutrition. Detecting IUGR is one of the main aims of routine ultrasound screening in antenatal care. A study found that the sensitivity of routine ultrasound examinations in Germany was extremely low; merely 30% of cases with intrauterine growth retardation were diagnosed antenatally [35]. Further research is needed to validate our indicative findings regarding a higher risk of stillbirth in association with fetal malnutrition or IUGR association among women from the Middle East and North Africa, and to examine whether differences in sensitivity of antenatal diagnostic investigation between women with and without migrant background exist.

Higher risks of stillbirth in some migrant groups might be associated with a more unfavourable use of antenatal care compared to women without migrant background, but it might also be assumed that there are differences in the quality of antenatal care. In this context, studies found that 25-30% of perinatal deaths are due to factors of suboptimal care [36,37]. Among the commonly found explanatory factors for this are the missed or delayed diagnosis of intrauterine growth retardation and an inadequate management of this condition, as well as the patient's non-compliance or delayed attendance to antenatal care [36,38]. A Europe-wide study ("Perinatal Death Audit Study") found that migrant women have a higher risk for perinatal mortality due to suboptimal factors during antenatal care [39]. Some of these factors seem to be associated with poor language skills leading to miscommunication between migrants and health providers, as well as a lack of knowledge and attention to cultural-related issues among maternity care professionals [40]. Researchers in Germany have found differences in the quality of antenatal care between women of German and non-German nationality [41]. The authors used the quality of documentation in the maternity record as a proxy indicator for the quality of care. Completeness of anamnestic data and documentation of consultation were lower among foreign women than among women without migrant background. The documentation of prenatal diagnoses (e.g. IUGR) is thus likely to be lower among women with migrant background and may result in insufficient surveillance of intrauterine growth.

Stillbirth and low birthweight relative to the gestational age can also be associated with congenital anomalies. We were not able to show considerable differences in the rate of congenital anomalies or modifications of risk of stillbirth adjusted for anomalies. However, several studies showed a higher perinatal mortality among newborns of women from non-western countries in the Netherlands and among Pakistani women in Great Britain, associated with a higher rate of congenital anomalies in these migrant groups [19,20,42]. A Norwegian study found that nearly 30% of infant deaths among Pakistani migrants were due to the high percentage of consanguine marriages in this group [20], with consanguinity as a risk factor e.g. for congenital anomalies. The prevalence of consanguine marriages is high among Turkish migrants, the largest migrant group by nationality in Germany. An association with higher rates of stillbirth might be probable [43,44]. However, this remains an assumption because the German perinatal database does not contain valid information on the parents' familial relationship.

### Strengths and Limitations

The strengths of this study are the large number of cases, its representativeness, the topicality and the completeness of data. We used population based and Germany-wide data. The data used were collected in a systematic way so we could confidently rule out selection bias. Furthermore, the study is the first to analyse relative differences in stillbirth rate and risk of stillbirth between several migrant groups in Germany.

The study, however, has its limitations. Although completeness of data is very high, the quality of some data items is sub-optimal. Jahn and Berle [45] were able to show that (obstetric) risk factors in the perinatal database are not documented as well as in the maternity log. Thus, there seems to be a loss of information in the data transmission process. "Occupation of the mother" showed a high non-response, and information on the father's occupation and income is lacking. Although smoking had a high non-response, it was included in the analyses because there was no evidence for a differential proportion of missings among women with and without migrant background. We are also not sure about the completeness of data concerning pre-eclampsia. While the eclampsia rate of 0.1% is similar to prior reported incidences for Germany [46,47], the pre-eclampsia rate seems to be too low, especially among migrant women. Prior studies reported pre-eclampsia incidences of 5-10% for Germany [47]. Eclampsia and pre-eclampsia occur during pregnancy and are mostly diagnosed and treated outpatiently by a gynaecologist. A possible reason for the inadequate reporting of these in the BQS data might therefore be that information from the maternal card about outpatient diagnoses and treatments are insufficiently transferred to the hospital data and subsequently to the BQS. Birth related factors showed a high completeness.

One of the major limitations in the conduction of migration- and socioeconomic-sensitive research in the field of obstetrics in Germany is the lack of appropriate variables of migrant status and socioeconomic status collected in the perinatal databases. As there is no clear definition of "region of origin" in the instructions for completing the perinatal sheet in German hospitals, we cannot be sure about how the variable is collected in hospitals. Hence, our data set might include more than one migrant generation without opportunity for differentiation. Further, the combination of several countries in one group is unsatisfactory. Each group includes different national, ethnic and cultural populations that are not at all homogenous. This makes the interpretation of our results more difficult. While it can be assumed that women of Turkish origin represent the largest proportion in the group "Middle East/North Africa", we cannot rule out that our conclusions do not apply to the other migrant groups included. In addition, the group "Asia" is not further defined, making it difficult to draw conclusions about this group from the results presented in this paper. There might be huge differences in the group of Asian migrants especially regarding stillbirth rates as Asia includes some of the most deprived areas of the world such as Central Asia with high stillbirth rates, and some of the lowest stillbirth rates, e.g. in Japan. Furthermore, the indicator of socioeconomic status in the database is not optimal as it only provides information about the mother's occupation. Further information, e.g. concerning the maternal education level as well as the father's education level and occupation, would be necessary to describe and study the effect of SES on birth outcomes among migrants in a more appropriate manner. As the perinatal database is the only available routine database for perinatal research in Germany, the implementation of appropriate variables for migrant status (at least country of birth of father and mother as well as country of birth of mother's parents and nationality) and the socioeconomic status comprises a major challenge for the future. The perinatal database was mainly developed for, and is still an instrument for quality control in German hospitals. Hence its use for routine health reporting or health research is limited. A change of data collection and implementation of new variables is highly dependent on the backup from politics in each federal state in Germany, and would thus take a very long time. Nevertheless, there are already successfully working birth registries implemented in single regions in Germany, e.g. the Mainz birth defect monitoring system (called the ''Mainz Model"), which is based on an expanded perinatal sheet focussing mainly on anomalies. These kind of approaches should be promoted and expanded to other federal states in Germany.

## Conclusion

This study is the first in Germany to use a large nationwide perinatal dataset for the investigation of variations in the risk of stillbirth in relation to maternal migration background. It shows differences in relative risk of stillbirth among women of different regions of origin compared with women originating from Germany, with a significantly higher risk among women from the Middle East and North Africa (which includes Turkey). This migrant group also showed by far the highest proportion of low social status mothers. Our findings lend support to planned efforts to develop and evaluate culturally sensitive health promotion and prevention programmes for pregnant women with low education and a Turkish migrant background in Germany. Considerable differences concerning obstetric risk factors, e.g. preterm birth, low birthweight as well as number and timing of antenatal care visits, could not be observed.

Our findings suggest a higher risk of stillbirth among women with migrant background due to intrauterine growth retardation. Differences in the quality of provision of antenatal care and/or the management of conditions identified cannot be ruled out. Migrant women with an early uptake and a sufficient number of antenatal visits experienced a higher risk of stillbirth. This indicates a particular need to closely monitor risk pregnancies among these women. Further studies should investigate whether the higher risk of stillbirths among migrant women is due to a lower sensitivity of antenatal screening for intrauterine growth retardation and insufficient follow-up interventions.

## Competing interests

Funding for these analyses was granted by the Federal Association of Local Health Insurance Funds [AOK-Bundesverband]. AR was financed from this grant during the study period. The AOK-Bundesverband had no influence on the data analysis and interpretation.

## Authors' contributions

AR, OR and JS conceived the study. AR performed the data analysis and wrote the first draft. MK gave statistical advice and coordinated the data analysis at BQS. JS participated in the data analysis. JS and OR supported the interpretation of results. All authors helped to draft the manuscript and read and approved it in its final form.

## Pre-publication history

The pre-publication history for this paper can be accessed here:

http://www.biomedcentral.com/1471-2393/11/63/prepub

## Supplementary Material

Additional file 1 **Table S1 - Overview of countries in the different regions of origin, according to BQS Institute**. This table gives an overview of countries that are included in the variable "regions of origin", according to BQS Institute. Click here for file

Additional file 2 **Table S2 - Further characteristics of study population by mother's region of origin (in %), 2004-2007, (N = 2,623,064)**. Further characteristics of study population by mother's region of origin that are not shown in Table 1. Table S3 - Crude and stratified stillbirth rates and appropriate relative mortality risks according to mother's region of origin. This table shows stillbirth rates and relative mortality risks stratified for mother's region of origin as well as further socio-demographic, lifestyle-related and obstetric factors. Click here for file
