# Supplementary material for: Stillbirth differences according to regions of origin: an analysis of the German perinatal database, 2004-2007
Source: BMC Pregnancy Childbirth. 2011 Sep 21;11:63. doi: 10.1186/1471-2393-11-63 (PMC3188470; doi:10.1186/1471-2393-11-63)
Supplement: Additional file 1 — Table S1 - Overview of countries in the different regions of origin, according to BQS Institute. This table gives an overview of countries that are included in the variable "regions of origin", according to BQS Institute. [file 1471-2393-11-63-S1.DOC]

**Additional file 1**

**Table S1 - Overview of countries in the different regions of origin,**

according to BQS Institute.

|  | **Region of origin** | **Included countries** |
| --- | --- | --- |
| 1 | Middle and Northern Europe, North America | Austria, Switzerland, France, Belgium, The Netherlands, Luxembourg, Great Britain, Denmark, Sweden, Norwegian, Finland, USA |
| 2 | Mediterranean countries | Former Yugoslavia, Greece, Italy, Spain, Portugal, Israel, Malta, Cyprus |
| 3 | Eastern Europe | Former Soviet Union, Poland, Czechia, Slovakia, Romania, Bulgaria, Hungary |
| 4 | Middle East | Turkey, Afghanistan, Pakistan, North Africa incl. Arab countries |
| 5 | Asia | Excl. 4 |
