# Supplementary material for: Stillbirth differences according to regions of origin: an analysis of the German perinatal database, 2004-2007
Source: BMC Pregnancy Childbirth. 2011 Sep 21;11:63. doi: 10.1186/1471-2393-11-63 (PMC3188470; doi:10.1186/1471-2393-11-63)
Supplement: Additional file 2 — Table S2 - Further characteristics of study population by mother's region of origin (in %), 2004-2007, (N = 2,623,064). Further characteristics of study population by mother's region of origin that are not shown in Table 1. Table S3 - Crude and stratified stillbirth rates and appropriate relative mortality risks according to mother's region of origin. This table shows stillbirth rates and relative mortality risks stratified for mother's region of origin as well as further socio-demographic, lifestyle-related and obstetric factors. [file 1471-2393-11-63-S2.DOC]

|  | **Germany** | **Middle and Northern Europe, North America** | **Medi-terranean** | **Eastern Europe** | **Middle East, North Africa** | **Asia** | **p value*** |
| --- | --- | --- | --- | --- | --- | --- | --- |
| **Number of births** | 2,166,005 | 37,854 | 85,874 | 138,217 | 164,341 | 30,773 |  |
| **Sex of newborn** |  |  |  |  |  |  |  |
| Male | 51.2 | 50.8 | 51.5 | 51.2 | 51.1 | 51.0 | 0.094 |
| Female | 48.8 | 49.2 | 48.5 | 48.8 | 48.9 | 49.0 |  |
| **High Parity (>4 children)** |  |  |  |  |  |  |  |
| No | 99.3 | 99.3 | 98.4 | 98.4 | 97.5 | 99.4 | <0.0001 |
| Yes | 0.7 | 0.7 | 1.6 | 1.6 | 2.5 | 0.6 |  |
| **Birth weight (g)** |  |  |  |  |  |  |  |
| 500-999 | 0.7 | 0.7 | 0.7 | 0.7 | 0.8 | 0.6 | <0.0001 |
| 1000-1499 | 0.8 | 0.8 | 0.7 | 0.7 | 0.8 | 0.8 |  |
| 1500-1999 | 1.5 | 1.3 | 1.4 | 1.2 | 1.4 | 1.4 |  |
| 2000-2499 | 4.4 | 3.8 | 4.2 | 3.4 | 4.1 | 4.5 |  |
| 2500-2999 | 16.1 | 15.2 | 16.8 | 13.8 | 16.5 | 19.4 |  |
| 3000-3499 | 36.6 | 36.9 | 38.2 | 36.1 | 38.4 | 40.3 |  |
| 3500+ | 40.3 | 41.5 | 38.4 | 44.4 | 38.3 | 33.3 |  |
| **Gestational age (weeks)** |  |  |  |  |  |  |  |
| < 28 | 0.4 | 0.5 | 0.5 | 0.5 | 0.6 | 0.4 | <0.0001 |
| 28–31 | 0.8 | 0.7 | 0.7 | 0.7 | 0.8 | 0.7 |  |
| 32–36 | 5.0 | 4.5 | 4.4 | 4.2 | 4.5 | 4.6 |  |
| 37–41 | 82.2 | 82.9 | 83.7 | 81.8 | 83.4 | 85.6 |  |
| ≥ 42 | 11.6 | 11.4 | 10.6 | 12.8 | 10.7 | 8.7 |  |
| **Number of ultrasounds** |  |  |  |  |  |  |  |
| 0-2 | 3.8 | 7.2 | 6.4 | 7.3 | 6.0 | 7.5 | <0.0001 |
| 3-4 | 49.9 | 46.2 | 53.0 | 54.0 | 54.4 | 51.8 |  |
| >4 | 38.3 | 36.1 | 31.3 | 31.2 | 32.5 | 32.2 |  |
| **First ultrasound (gestational week)** |  |  |  |  |  |  |  |
| 0-8 | 21.2 | 18.6 | 18.4 | 17.3 | 18.5 | 16.2 | <0.0001 |
| 9-12 | 57.3 | 50.5 | 53.1 | 53.6 | 55.7 | 51.9 |  |
| 13-16 | 10.5 | 11.8 | 12.6 | 12.9 | 12.9 | 13.6 |  |
| >16 | 4.4 | 10.3 | 7.9 | 10.2 | 7.2 | 11.5 |  |
| **Umbilical artery pH** |  |  |  |  |  |  |  |
| <7.1 | 1.6 | 1.5 | 1.3 | 1.3 | 1.5 | 1.4 | <0.0001 |
| ≥7.1 | 96.4 | 96.3 | 96.8 | 96.7 | 96.6 | 96.6 |  |
| **Preeclampsia (birth risk)** |  |  |  |  |  |  |  |
| No | 97.5 | 98.3 | 98.5 | 98.3 | 98.7 | 98.8 | <0.0001 |
| Yes | 2.5 | 1.7 | 1.5 | 1.7 | 1.3 | 1.2 |  |
| **Eclampsia (complication during delivery)** |  |  |  |  |  |  |  |
| No | 99.9 | 99.9 | 99.9 | 99.9 | 99.9 | 99.9 | 0.166 |
| Yes | 0.1 | 0.1 | 0.1 | 0.1 | 0.1 | 0.1 |  |
| **Placenta praevia (pregnancy risk)** |  |  |  |  |  |  |  |
| No | 99.7 | 99.8 | 99.8 | 99.8 | 99.7 | 99.5 | <0.0001 |
| Yes | 0.3 | 0.2 | 0.2 | 0.2 | 0.3 | 0.5 |  |
| **Placenta praevia (birth risk)** |  |  |  |  |  |  |  |
| No | 99.6 | 99.7 | 99.7 | 99.7 | 99.7 | 99.3 | <0.0001 |
| Yes | 0.4 | 0.3 | 0.3 | 0.3 | 0.3 | 0.7 |  |

**Table S2 - Further characteristics of study population by mother’s region of origin (in %), 2004-2007, (N = 2,623,064)**

* p value for a chi-square test

**Table S3 -** Crude and stratified stillbirth rates and appropriate relative mortality risks according to mother’s region of origin

|  | **Germany** | | **Middle and Northern Europe. North America** | | | | **Mediterranean countries** | | | | **Eastern Europe** | | | | **Middle East. North Africa (incl. Turkey)** | | | | **Asia** | | | |
| --- | --- | --- | --- | --- | --- | --- | --- | --- | --- | --- | --- | --- | --- | --- | --- | --- | --- | --- | --- | --- | --- | --- |
|  | **Num-ber** | **Rate*** | **Num-ber** | **Rate*** | **RR** | **95 % CI** | **Num-**  **ber** | **Rate*** | **RR** | **95 % CI** | **Num-**  **ber** | **Rate*** | **RR** | **95 % CI** | **Num-**  **ber** | **Rate*** | **RR** | **95 % CI** | **Num-**  **ber** | **Rate*** | **RR** | **95 % CI** |
| **Total** | **6,865** | **3.17** | **111** | **2.93** | **0.92** | **0.76-1.11** | **309** | **3.60** | **1.14** | **1.02-1.28** | **458** | **3.31** | **1.04** | **0.95-1.14** | **700** | **4.26** | **1.34** | **1.24-1.45** | **115** | **3.74** | **1.18** | **0.98-1.42** |
| **Sex of newborn** |  |  |  |  |  |  |  |  |  |  |  |  |  |  |  |  |  |  |  |  |  |  |
| Male | 3,594 | 3.24 | 56 | 2.91 | 0.90 | 0.69-1.17 | 157 | 3.55 | 1.09 | 0.93-1.28 | 238 | 3.36 | 1.04 | 0.91-1.18 | 369 | 4.39 | **1.36** | **1.22-1.51** | 66 | 4.20 | **1.30** | **1.02-1.65** |
| Female | 3,271 | 3.09 | 55 | 2.95 | 0.95 | 0.73-2.14 | 152 | 3.65 | 1.18 | 1.00-1.39 | 220 | 3.26 | 1.05 | 0.92-1.21 | 331 | 4.12 | **1.33** | **1.19-1.49** | 49 | 3.25 | 1.05 | 0.79-1.39 |
| **Single status** |  |  |  |  |  |  |  |  |  |  |  |  |  |  |  |  |  |  |  |  |  |  |
| No | 4,898 | 2.95 | * | * | * | * | 243 | 3.28 | 1.11 | 0.98-1.27 | 368 | 3.12 | 1.06 | 0.95-1.18 | 624 | 4.22 | **1.43** | **1.32-1.55** | * | * | * | * |
| Yes | 1,282 | 3.97 | * | * | * | * | 35 | 5.97 | **1.50** | **1.07-2.10** | 57 | 4.89 | 1.23 | 0.94-1.60 | 34 | 5.19 | 1.31 | 0.93-1.84 | * | * | * | * |
| **Social status** |  |  |  |  |  |  |  |  |  |  |  |  |  |  |  |  |  |  |  |  |  |  |
| Low | 2,341 | 3.40 | 48 | 2.79 | 0.82 | 0.62-1.09 | 195 | 3.80 | 1.12 | 0.97-1.29 | 246 | 3.17 | 0.93 | 0.82-1.06 | 514 | 4.38 | **1.29** | **1.17-1.42** | * | * | * | * |
| Middle | 2,266 | 2.77 | 24 | 2.63 | 0.95 | 0.64-1.42 | 29 | 2.08 | 0.75 | 0.52-1.08 | 89 | 2.98 | 1.08 | 0.87-1.33 | 46 | 2.92 | 1.05 | 0.79-1.41 | * | * | * | * |
| High | 652 | 2.24 | 16 | 3.42 | 1.53 | 0.93-2.51 | 7 | 1.87 | 0.83 | 0.40-1.75 | 22 | 3.08 | 1.38 | 0.90-2.11 | 11 | 3.07 | 1.37 | 0.76-2.49 | * | * | * | * |
| **Maternal age (years)** |  |  |  |  |  |  |  |  |  |  |  |  |  |  |  |  |  |  |  |  |  |  |
| <30 | 2,976 | 3.11 | 43 | 2.66 | 0.86 | 0.63-1.16 | 175 | 3.61 | 1.16 | 1.00-1.35 | 250 | 2.96 | 0.95 | 0.84-1.08 | 391 | 4.00 | **1.29** | **1.16-1.43** | 48 | 3.45 | 1.11 | 0.84-1.48 |
| 30-34 | 1,947 | 2.88 | 30 | 2.45 | 0.85 | 0.59-1.22 | 80 | 3.30 | 1.14 | 0.92-1.43 | 132 | 3.65 | **1.27** | **1.06-1.51** | 183 | 4.31 | **1.49** | **1.28-1.74** | 36 | 3.80 | 1.32 | 0.95-1.83 |
| ≥35 | 1,942 | 3.63 | 38 | 4.00 | 1.10 | 0.80-1.52 | 54 | 4.10 | 1.13 | 0.86-1.48 | 76 | 4.30 | 1.18 | 0.94-1.49 | 126 | 5.23 | **1.44** | **1.20-1.72** | 31 | 4.18 | 1.15 | 0.81-1.64 |
| **Maternal smoking (during pregnany)** |  |  |  |  |  |  |  |  |  |  |  |  |  |  |  |  |  |  |  |  |  |  |
| No | 4,443 | 2.81 | * | * | * | * | 165 | 2.84 | 1.01 | 0.86-1.18 | * | * | * | * | 461 | 3.82 | **1.36** | **1.23-1.50** | * | * | * | * |
| Yes | 970 | 4.10 | * | * | * | * | 44 | 4.58 | 1.12 | 0.83-1.51 | * | * | * | * | 75 | 4.96 | 1.21 | 0.96-1.53 | * | * | * | * |
| **Parity** |  |  |  |  |  |  |  |  |  |  |  |  |  |  |  |  |  |  |  |  |  |  |
| 0 | 2,846 | 3.09 | 51 | 3.75 | 1.21 | 0.92-1.60 | 110 | 3.89 | **1.26** | **1.04-1.52** | 171 | 3.47 | 1.12 | 0.96-1.31 | 206 | 4.27 | **1.38** | **1.20-1.59** | 36 | 3.25 | 1.05 | 0.76-1.46 |
| 1 | 1,986 | 2.85 | 34 | 2.69 | 0.94 | 0.67-1.32 | 83 | 3.02 | 1.06 | 0.85-1.32 | 119 | 2.68 | 0.94 | 0.78-1.13 | 171 | 3.54 | **1.24** | **1.06-1.45** | 38 | 3.66 | 1.28 | 0.93-1.77 |
| ≥2 | 2,033 | 3.70 | 26 | 2.24 | 0.60 | 0.41-0.89 | 116 | 3.86 | 1.04 | 0.87-1.26 | 168 | 3.78 | 1.02 | 0.87-1.20 | 323 | 4.76 | **1.29** | **1.15-1.45** | 41 | 4.39 | 1.19 | 0.87-1.62 |
| **High parity**  **(>4 children)** |  |  |  |  |  |  |  |  |  |  |  |  |  |  |  |  |  |  |  |  |  |  |
| No | 6,769 | 3.15 | * | * | * | * | 297 | 3.52 | 1.12 | 0.99-1.25 | 446 | 3.28 | 1.04 | 0.95-1.15 | 672 | 4.19 | **1.33** | **1.23-1.44** | * | * | * | * |
| Yes | 96 | 6.21 | * | * | * | * | 12 | 8.70 | 1.40 | 0.77-2.55 | 12 | 5.60 | 0.90 | 0.50-1.64 | 28 | 6.79 | 1.09 | 0.72-1.66 | * | * | * | * |
| **Multiple birth** |  |  |  |  |  |  |  |  |  |  |  |  |  |  |  |  |  |  |  |  |  |  |
| Singletons | 6,294 | 3.01 | 99 | 2.71 | 0.90 | 0.74-1.10 | 296 | 3.56 | **1.18** | **1.05-1.33** | 412 | 3.06 | 1.02 | 0.92-1.12 | 653 | 4.10 | **1.36** | **1.26-1.48** | 108 | 3.59 | 1.19 | 0.99-1.44 |
| Twins or more | 571 | 7.78 | 12 | 9.48 | 1.22 | 0.69-2.15 | 13 | 4.94 | 0.63 | 0.37-1.10 | 46 | 12.40 | **1.59** | **1.18-2.15** | 47 | 9.51 | 1.22 | 0.91-1.64 | 7 | 10.26 | 1.32 | 0.63-2.77 |
| **Gestational age**  **(weeks)^a^** |  |  |  |  |  |  |  |  |  |  |  |  |  |  |  |  |  |  |  |  |  |  |
| < 28 | 1782 | 188.89 | 37 | 202.19 | 1.07 | 0.80-1.43 | 63 | 159.09 | 0.84 | 0.67-1.06 | 107 | 154.85 | 0.82 | 0.69-0.98 | 154 | 167.94 | 0.89 | 0.77-1.03 | 25 | 200.00 | 1.06 | 0.74-1.51 |
| 28-31 | 1189 | 72.86 | 15 | 57.25 | 0.79 | 0.48-1.29 | 55 | 88.00 | 1.21 | 0.93-1.56 | 84 | 85.37 | 1.17 | 0.95-1.45 | 129 | 97.65 | **1.34** | **1.13-1.59** | 14 | 63.93 | 0.88 | 0.53-1.46 |
| 32-36 | 1,596 | 14.75 | 27 | 15.90 | 1.08 | 0.74-1.57 | 78 | 20.41 | **1.38** | **1.10-1.73** | 119 | 20.47 | **1.39** | **1.15-1.67** | 187 | 25.08 | **1.70** | **1.46-1.97** | 30 | 21.31 | **1.44** | **1.01-2.06** |
| ≥ 37 | 2,295 | 1.13 | 32 | 0.90 | 0.79 | 0.56-1.12 | 112 | 1.38 | **1.22** | **1.01-1.48** | 148 | 1.13 | 1.00 | 0.85-1.18 | 230 | 1.49 | **1.32** | **1.15-1.51** | 46 | 1.59 | **1.40** | **1.05-1.88** |
| **Birthweight (g)^b^** |  |  |  |  |  |  |  |  |  |  |  |  |  |  |  |  |  |  |  |  |  |  |
| 500-999 | 2,313 | 195.32 | 39 | 178.90 | 0.92 | 0.69-1.22 | 98 | 206.32 | 1.06 | 0.88-1.26 | 144 | 184.85 | 0.95 | 0.81-1.10 | 217 | 208.05 | 1.07 | 0.94-1.21 | 29 | 198.63 | 1.02 | 0.73-1.41 |
| 1000-1499 | 905 | 57.22 | 21 | 79.25 | 1.38 | 0.91-2.10 | 40 | 72.46 | 1.27 | 0.93-1.72 | 72 | 77.42 | **1.35** | **1.07-1.70** | 95 | 76.61 | **1.34** | **1.09-1.64** | 18 | 82.95 | 1.45 | 0.93-2.27 |
| 1500-1999 | 820 | 26.22 | 9 | 17.96 | 0.69 | 0.36-1.31 | 33 | 29.18 | 1.11 | 0.79-1.57 | 63 | 39.87 | **1.52** | **1.18-1.96** | 78 | 34.71 | **1.32** | **1.05-1.66** | 13 | 31.18 | 1.19 | 0.69-2.04 |
| 2000-2499 | 799 | 8.46 | 8 | 5.60 | 0.66 | 0.33-1.33 | 38 | 10.76 | 1.27 | 0.92-1.76 | 54 | 11.63 | **1.37** | **1.05-1.81** | 123 | 18.59 | **2.20** | **1.82-2.65** | 14 | 10.12 | 1.20 | 0.71-2.02 |
| 2500-2999 | 845 | 2.43 | 12 | 2.09 | 0.86 | 0.48-1.51 | 44 | 3.05 | 1.25 | 0.93-1.70 | 47 | 2.47 | 1.02 | 0.76-1.36 | 67 | 2.47 | 1.02 | 0.79-1.30 | 14 | 2.35 | 0.97 | 0.57-1.64 |
| 3000-3499 | 695 | 0.88 | 16 | 1.15 | 1.31 | 0.80-2.14 | 38 | 1.16 | 1.32 | 0.95-1.83 | 40 | 0.80 | 0.91 | 0.66-1.26 | 61 | 0.97 | 1.10 | 0.85-1.43 | 16 | 1.29 | 1.47 | 0.90-2.42 |
| ≥ 3500 | 488 | 0.56 | 6 | 0.38 | 0.68 | 0.31-1.53 | 18 | 0.55 | 0.98 | 0.61-1.56 | 38 | 0.62 | 1.11 | 0.80-1.54 | 59 | 0.94 | **1.67** | **1.28-2.19** | 11 | 1.07 | **1.92** | **1.06-3.49** |
| **Gestational age (weeks) & birth weight (g)** |  |  |  |  |  |  |  |  |  |  |  |  |  |  |  |  |  |  |  |  |  |  |
| < 37 & < 2500 | 4,330 | 44.92 | 72 | 46.36 | 1.03 | 0.82-1.30 | * | * | * | * | 300 | 56.69 | **1.26** | **1.13-1.41** | 454 | 64.22 | **1.43** | **1.30-1.57** | * | * | * | * |
| ≥ 37 & < 2500 | 504 | 8.62 | 5 | 5.67 | 0.66 | 0.27-1.58 | * | * | * | * | 33 | 12.00 | 1.39 | 0.98-1.98 | 59 | 14.04 | **1.63** | **1.25-2.13** | * | * | * | * |
| < 37 & ≥ 2500 | 237 | 6.33 | 7 | 11.86 | 1.87 | 0.89-3.96 | * | * | * | * | 10 | 4.56 | 0.72 | 0.38-1.35 | 16 | 6.17 | 0.98 | 0.59-1.62 | * | * | * | * |
| ≥ 37 & ≥ 2500 | 1,791 | 0.91 | 27 | 0.78 | 0.85 | 0.58-1.25 | * | * | * | * | 115 | 0.90 | 0.99 | 0.82-1.20 | 171 | 1.14 | **1.25** | **1.07-1.47** | * | * | * | * |
| **Number of antenatal visits** |  |  |  |  |  |  |  |  |  |  |  |  |  |  |  |  |  |  |  |  |  |  |
| 0-4 | 911 | 30.00 | 13 | 13.33 | **0.44** | **0.26-0.77** | 46 | 19.42 | **0.65** | **0.48-0.87** | 75 | 17.31 | **0.58** | **0.46-0.73** | 103 | 29.10 | 0.97 | 0.79-1.19 | 20 | 19.76 | 0.66 | 0.42-1.02 |
| 5 bis 7 | 1,980 | 21.43 | 40 | 15.32 | **0.71** | **0.52-0.98** | 89 | 13.19 | **0.62** | **0.50-0.76** | 131 | 12.18 | **0.57** | **0.48-0.68** | 216 | 16.91 | 0.79 | 0.69-0.91 | 34 | 12.23 | 0.57 | 0.41-0.80 |
| 8 bis 11 | 2,157 | 2.31 | 23 | 1.44 | **0.62** | **0.41-0.94** | 92 | 2.27 | 0.98 | 0.80-1.21 | 139 | 2.14 | 0.93 | 0.78-1.10 | 219 | 2.67 | 1.15 | 1.00-1.33 | 39 | 2.57 | 1.11 | 0.81-1.52 |
| ≥12 | 907 | 0.97 | 16 | 1.12 | 1.15 | 0.70-1.88 | 31 | 1.10 | 1.13 | 0.79-1.61 | 44 | 0.92 | 0.94 | 0.70-1.28 | 74 | 1.37 | **1.41** | **1.11-1.78** | 9 | 1.00 | 1.03 | 0.53-1.98 |
| **First antenatal visit (gestational week)** |  |  |  |  |  |  |  |  |  |  |  |  |  |  |  |  |  |  |  |  |  |  |
| <9. | 2,834 | 2.81 | * | * | * | * | 140 | 3.86 | **1.37** | **1.16-1.63** | 140 | 2.55 | 0.91 | 0.76-1.07 | 290 | 3.93 | **1.40** | **1.24-1.58** | 46 | 4.18 | **1.48** | **1.11-1.99** |
| 9.-12. | 2,371 | 2.92 | * | * | * | * | 86 | 2.81 | 0.96 | 0.78-1.19 | 175 | 3.43 | **1.17** | **1.01-1.37** | 238 | 4.03 | **1.38** | **1.21-1.57** | 40 | 3.42 | 1.17 | 0.86-1.60 |
| 13.-16. | 426 | 3.44 | * | * | * | * | 16 | 2.43 | 0.71 | 0.43-1.16 | 31 | 2.55 | 0.74 | 0.51-1.07 | 51 | 4.08 | 1.19 | 0.89-1.58 | 6 | 2.05 | 0.59 | 0.27-1.33 |
| ≥17. | 367 | 5.42 | * | * | * | * | 23 | 4.55 | 0.84 | 0.55-1.28 | 48 | 4.23 | 0.78 | 0.58-1.05 | 41 | 4.98 | 0.92 | 0.67-1.27 | 11 | 3.85 | 0.71 | 0.39-1.29 |
| **Number of ultrasounds** |  |  |  |  |  |  |  |  |  |  |  |  |  |  |  |  |  |  |  |  |  |  |
| 0-2 | 1,255 | 15.09 | 23 | 8.48 | **0.56** | **0.37-0.85** | 62 | 11.26 | **0.75** | **0.58-0.96** | 83 | 8.27 | 0.55 | 0.44-0.68 | 132 | 13.44 | 0.89 | 0.75-1.06 | 20 | 8.68 | 0.58 | 0.37-0.89 |
| 3-4 | 2,702 | 2.50 | 42 | 2.40 | 0.96 | 0.71-1.30 | 122 | 2.68 | 1.07 | 0.89-1.29 | 192 | 2.57 | 1.03 | 0.89-1.19 | 301 | 3.37 | **1.35** | **1.20-1.52** | 55 | 3.45 | **1.38** | **1.06-1.80** |
| ≥5 | 2,015 | 2.43 | 30 | 2.19 | 0.90 | 0.63-1.29 | 73 | 2.72 | 1.12 | 0.89-1.41 | 109 | 2.53 | 1.04 | 0.86-1.26 | 179 | 3.35 | **1.38** | **1.18-1.61** | 29 | 2.93 | 1.21 | 0.84-1.74 |
| **First ultrasound (gestational week)** |  |  |  |  |  |  |  |  |  |  |  |  |  |  |  |  |  |  |  |  |  |  |
| 0-8 | 2,834 | 2.81 | * | * | * | * | 140 | 3.86 | **1.37** | **1.16-1.63** | 140 | 2.55 | 0.91 | 0.76-1.07 | 290 | 3.93 | **1.40** | **1.24-1.58** | 46 | 4.18 | **1.48** | **1.11-1.99** |
| 9-12 | 2,371 | 2.92 | * | * | * | * | 86 | 2.81 | 0.96 | 0.78-1.19 | 175 | 3.43 | **1.17** | **1.01-1.37** | 238 | 4.03 | **1.38** | **1.21-1.57** | 40 | 3.42 | 1.17 | 0.86-1.60 |
| 13-16 | 426 | 3.44 | * | * | * | * | 16 | 2.43 | 0.71 | 0.43-1.16 | 31 | 2.55 | 0.74 | 0.51-1.07 | 51 | 4.08 | 1.19 | 0.89-1.58 | 6 | 2.05 | 0.59 | 0.27-1.33 |
| ≥17 | 367 | 5.42 | * | * | * | * | 23 | 4.55 | 0.84 | 0.55-1.28 | 48 | 4.23 | 0.78 | 0.58-1.05 | 41 | 4.98 | 0.92 | 0.67-1.27 | 11 | 3.85 | 0.71 | 0.39-1.29 |
| **Congenital malformation** |  |  |  |  |  |  |  |  |  |  |  |  |  |  |  |  |  |  |  |  |  |  |
| No | 5,863 | 2.73 | 98 | 2.62 | 0.96 | 0.78-1.17 | 263 | 3.10 | 1.13 | 1.00-1.28 | 387 | 2.83 | 1.03 | 0.93-1.15 | 594 | 3.65 | **1.34** | **1.23-1.45** | 101 | 3.32 | 1.21 | 1.00-1.48 |
| Yes | 1,002 | 45.58 | 13 | 31.10 | 0.68 | 0.40-1.17 | 46 | 46.51 | 1.02 | 0.76-1.36 | 71 | 48.46 | 1.06 | 0.84-1.34 | 106 | 63.63 | **1.40** | **1.15-1.69** | 14 | 45.75 | 1.00 | 0.60-1.68 |
| **Pre-Eclampsia (birth risk)** |  |  |  |  |  |  |  |  |  |  |  |  |  |  |  |  |  |  |  |  |  |  |
| No | 6,750 | 3.20 | * | * | * | * | 303 | 3.58 | 1.12 | 1.00-1.26 | 450 | 3.31 | 1.04 | 0.94-1.14 | 693 | 4.27 | **1.34** | **1.24-1.45** | * | * | * | * |
| Yes | 115 | 2.15 | * | * | * | * | 6 | 4.55 | 2.12 | 0.93-4.80 | 8 | 3.33 | 1.55 | 0.76-3.16 | 7 | 3.24 | 1.51 | 0.70-3.23 | * | * | * | * |
